# When a Dust Storm Is Not a Dust Storm: Reliability of Dust Records From the Storm Events Database and Implications for Geohealth Applications

**DOI:** 10.1029/2022GH000699

**Published:** 2023-01-05

**Authors:** K. Ardon‐Dryer, T. E. Gill, D. Q. Tong

**Affiliations:** ^1^ Department of Geosciences Texas Tech University Lubbock TX USA; ^2^ Department of Earth, Environmental and Resource Sciences The University of Texas at El Paso El Paso TX USA; ^3^ Department of Atmospheric, Oceanic and Earth Sciences/Center for Spatial Information Science and Systems George Mason University Fairfax VA USA

**Keywords:** Storm Events Database, dust storm, blowing dust events, USA

## Abstract

Windblown dust impacts human health, air quality, and climate. The National Weather Service Storm Events Database (SED) is a widely used dataset of significant or unusual weather, including dust storms (DS), and resulting deaths, injuries, and material losses in the USA. The SED is frequently used by medical, social, and atmospheric scientists. However, it is uncertain whether this dataset reliably represents spatial and temporal variations and trends of DS. Analyzing the SED from 2000 to 2020 identified 1,167 DS reports; removing reports of the same event from multiple locations left 647 DS in 21 USA states. The number of DS ranged from 12 in 2008 to 53 in 2018, with no strong interannual trends detected (*R* ^2^ was 0.3). By examining the DS events reported in the SED based on meteorological observations including wind speed, visibility, and weather codes, we determined that the SED was not only missing many DS (visibility <1 km), but also included many blowing dust (BLDU) events. 49.9% of 491 reported DS events in SED had visibility >1 km and were incorrectly reported as DS. Underrepresentation of DS and inclusion of BLDU may be partially due to the diverse sources contributing to the SED and a lack of verification of the reports and their consistency. Although the SED is an extremely useful and valuable database of impactful weather, including DS, the issues found in this study warrant caution in use of this dataset for many geohealth applications.

## Key Points

- Storm Events Database, a widely used weather event database for many health effects studies, is lacking many dust storms (DS) events
- Although the Storm Database is representative and useful, it may not be comprehensive or complete and thus should be used carefully
- There is a need for a new database of dust events, that will include all levels of dust events (including blowing dust and DS)

## 1 Introduction

Dust storm (DS) is a meteorological phenomenon caused by wind erosion of soil/sediment or suspension of particles from the land surface into the air by mechanical means (Goudie, 2014; Middleton, 2017). Dust particles are one of the most important natural contributors to atmospheric particulate matter (PM; PM_2.5_ and PM_10_, particulate matter with an aerodynamic diameter <2.5 and 10 μm, respectively) (Ardon‐Dryer & Kelley, 2022; Kelley et al., 2020; Shahsavani et al., 2012). The increase of dust particles during dust events can affect solar radiation by absorbing and scattering the sun's radiation (Haywood et al., 2003), influence the atmospheric vertical electric field (Ardon‐Dryer et al., 2022), influence cloud formation (Ardon‐Dryer & Levin, 2014; Bangert et al., 2012), have detrimental effects on the global economy (Tozer & Leys, 2013), as well as impact human well‐being, safety, and health (Ardon‐Dryer et al., 2020; Goudie, 2014).

According to the World Meteorological Organization (WMO), a DS is defined when the horizontal visibility is reduced by dust in the air to less than 1 km (UNEP, 2016; WMO, 2019). The USA Department of Transportation Federal Aviation Administration (FAA), based on guidance provided by the WMO, also uses the same guidelines (FAA, 2022). Operational weather warning in the USA National Weather Service (NWS) adopts a more stringent criterion of visibility of 0.4 km (one fourth mile) or less to report or warn of a DS (NWS, 2022a). Blowing dust (BLDU) or widespread dust (DU) is reported by the NWS (OFCM, 1995) as a less severe dust event characterized by airborne dust with higher visibility values up to 11.3 km (7 miles). In the 1990s and early 2000s, human weather observers in the USA were largely replaced by Automated Surface Observing Stations (ASOS). The ASOS system sometimes reports aerosol‐related visibility degradation including dust simply as “haze” (HZ) (Bernier, 1995; Kelley & Ardon‐Dryer, 2021), defined by the NWS as aggregation in the atmosphere of very fine, widely dispersed, solid or liquid particles, or both, giving the air an opalescent appearance that subdues colors (Lee et al., 2012).

There is no clear consensus worldwide or in the USA regarding how dust occurrence should be identified for climatological or epidemiological studies (Rublee et al., 2020). Previous environmentally‐focused studies used various data records to examine the distribution of observed dust storms or dust events across the USA. Some used visibility from weather stations (Orgill & Sehmel, 1976), others identified dust events based on weather report codes (Kandakji et al., 2020; Kelley & Ardon‐Dryer, 2021; Xi, 2021), and some looked at changes of PM_10_ concentrations or the ratios between PM_10_, and PM_2.5_ concentrations measured by air quality monitoring sites (Lei & Wang, 2014; Tong et al., 2012), while others used ground‐based aerosol optical depth (AOD) measurements and satellite images (Lei et al., 2016; Pu et al., 2020; Tong et al., 2017). Some studies used a combination of different methods to identify dust events, such as weather report codes, wind speed, and visibility threshold in addition to changes in PM concentrations (Eagar et al., 2017), or a combination of weather report codes, AOD, and changes in PM (Lei et al., 2016).

Different studies that examined the impact of dust on human health in the USA, either focused on one region (Comrie, 2021; Grineski et al., 2011; Hefflin et al., 1994; Herrera‐Molina et al., 2021; Ostro et al., 1999) or the entire USA (Crooks et al., 2016; Jones, 2020; Rublee et al., 2020). In some studies, environmental observations such as wind speed, PM concentrations from nearby air quality monitors, and/or reported weather codes from nearby ASOS were used to identify dust events (Grineski et al., 2011; Herrera‐Molina et al., 2021), while others used the Storm Events Database (SED) as the source of their DS dataset (Crooks et al., 2016; Jones, 2020; Rublee et al., 2020).

The SED of the National Center for Environmental Information (NCEI) and the National Oceanic and Atmospheric Administration is the most widely used dataset of information on significant and/or unusual weather phenomena in the USA. This database is used to populate an official publication titled *Storm Data* (NCEI, 2022). According to NCEI, the SED includes data on the occurrence of storms and other significant weather phenomena having sufficient intensity to cause loss of life, injuries, significant property damage, and/or disruption to commerce. Rare, unusual, weather phenomena that generate media attention, such as snow flurries in South Florida or the San Diego, California coastal area are also included in the SED. Other significant meteorological events, such as record maximum or minimum temperatures or precipitation that occur in connection with another event, may also be included. The NWS reporting instructions includes a category of events in the SED named DS. Dust storms are defined as strong winds that lift dust or sand particles over dry ground that have little or no vegetation. These particles can reduce visibility below locally/regionally established values (usually one fourth mile or less), which could result in a fatality, injury, damage, or major disruption of transportation. If the event that occurred is considered significant, even though it affected a small area, it should be entered into the database (NWS, 2022b).

The SED has been widely used by researchers to examine extreme weather's impact on human health and safety. For example, in the atmospheric science literature, it was used to examine mortality rates due to heat and cold (Dixon et al., 2005), injuries and mortality due to lightning strikes (Ashley & Gilson, 2009; López et al., 1993), the impact of winter precipitation on fatalities (Black & Mote, 2015), as well as the associations of convective and non‐convective wind events on fatalities (Ashley & Black, 2008; Black & Ashley, 2010). The SED has been widely used by researchers in fields outside the geosciences to investigate correlations between dust storms in the USA and factors including mortality (Crooks et al., 2016), freeway safety (Mohebbi et al., 2019), intensive care unit admissions (Rublee et al., 2020), birth weight and premature birth rates (Jones, 2020), the incidence of Valley fever (Comrie, 2021), neighborhood‐scale public health impacts of air pollution (Lothrop et al., 2022), and violent crime (Jones, 2022). The SED has been proposed as the data source for events including dust storms for creating a hazard assessment for locating healthcare facilities (Skinner, 2010), and for creating a “natural disasters index” (Mahanama et al., 2022).

Following our recent work (Tong et al., 2022), we suspected that there were discrepancies in dust reporting in the SED, and became aware of how studies, especially those performed by researchers outside atmospheric science who may not be aware of the SED's constraints and/or the definition of “dust storm,” have used DS from SED to ascribe relationships between dust storms and sociological, health and safety effects. Therefore, we were motivated to perform an examination of dust records in these widely used databases to test their accuracy.

## 2 Materials and Methods

The SED, maintained by the NCEI, lists all reported severe or damaging meteorological events that occur across the USA including thunderstorms, tornadoes, hurricanes, derechos, winter storms, flash and river floods, hail, heavy rain, heat and cold waves, dust storms and many others. The database also includes events that were associated with deaths, injuries, and material (property and crop) losses. The SED data are gathered in several ways. One way is by using the NWS storm report logs. These reports are usually gathered during the event itself, but sometimes can be added a few days later. SED data come from many sources including agency/official personnel including law enforcement and government officials, emergency management officials, departments of highways, NWS damage surveys or employees, trained spotters, and official meteorological station reports, as well as from the public including broadcast media, newspapers, and social media (NCEI, 2022). There are 40 different potential reporting sources (NWS, 2022b). The reports are made for location and local time, with the location provided as state and county (region) in which the event took place. In some cases, additional or detailed information will be provided in the event/episode narratives including information on the location of the event (e.g., “reported between Wellton and Tacna,” “reduced visibility to less than one fourth mile in Pahrump,” etc.) or on the cause of the event (e.g., “A strong cold front brought high winds and DS conditions”). In this study, DS reports from the SED were downloaded from the National Center for Environmental Information SED as CSV files for all USA states (not including Alaska and Hawaii) from 1 January 2000 to 31 December 2020. All DS events were reported in local time.

To examine each of the SED DS events, an in‐depth analysis was performed for the entire database (for each event reported). Examples of selected locations (e.g., Lubbock and El Paso, Texas, several sites in Utah and Colorado as well as the greater Phoenix area in Arizona) will be presented. Identification of each DS event was based on meteorological information retrieved from the Meteorological Aerodrome Reports that provide 5‐min to hourly meteorological measurements (e.g., wind speed, visibility, and present weather code) collected by the ASOS that were downloaded for each DS event (for each state) that was reported in the SED. Observations and identification of dust events/storms were based on the method used in Kelley and Ardon‐Dryer (2021) which includes identification of times with strong wind speed (>6 m s^−1^), low visibility <1 km, and reports in the weather code (including DS, BLDU, HZ, etc.) from an ASOS that was in close proximity to the location report in the SED.

## 3 Results and Discussion

### 3.1 Temporal Variations of Dust Storms

A total of 1167 DS reports were identified from the SED from 1 January 2000 to 31 December 2020. The greatest number of reports was for Arizona with a total of 480 DS reports while only one DS event was reported for the states of Delaware, Indiana, Missouri, and Wisconsin. In most cases, there was only a single report per day, but there were many with multiple reports of a single event (from multiple sources and locations). As an example, the highest number of reports for one DS event was on 24 February 2001, in Oklahoma which had a total of 29 different reports from various sources and different counties. This creates a potential oversampling issue that becomes problematic if this dataset is used to derive long‐term dust trends related to number of dust storms.

To make sure an event was not reported multiple times, DS reports, for each state (combining reports from multiple counties and sources per event), were combined to represent a single event per state (1 day). In some cases, two different DS were reported with several hours gap between them. In these cases, the DS was counted as two separate events. The start and end times of each event per state were recorded and combined per event.

**Figure 1:**
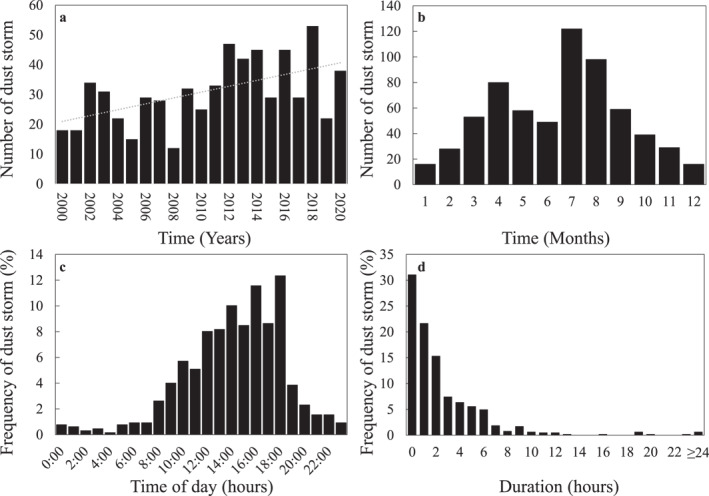
Temporal distribution and duration of dust storm events in the Contiguous United States: (a) yearly distribution, including trend line in gray; (b) monthly distribution; (c) time of day, and (d) duration, as reported in the Storm Events Database.

After removing multiple reports of the same event, a total of 647 DS events were reported in the SED from 2000 to 2020 across the USA. The number of annual DS reports ranged from 12 (in 2008) to 53 events (in 2018) (Figure 1a). No strong trend was found for the annual DS reports (*R* ^2^ was 0.3) but an increase in the overall number of DS reported was observed (slope was 1.0 per year). It is uncertain if this increase was a real increase in the number of dust storms over time or could have been caused by increases in population and weather awareness causing increased reporting (as suggested for tornadoes, as noted by Verbout et al. (2006) and summarized by Fricker et al. (2017)), as the number of reported dust storms also increases over time as shown in Figure S1 in Supporting Information S1. A bimodal distribution was observed for the monthly distribution (Figure 1b) with one peak in April with 80 DS events, and another stronger peak in July and August with 122 and 98 DS reports, respectively. Most of the DS were reported between 12:00 and 18:00 local time, the highest number of reports (12%) was at 18:00 (Figure 1c). Most of the DS events (30%) reported lasted an hour or less (Figure 1d), yet since the calculation of each DS duration was based solely on the reports received and entered into the SED, it is possible to assume that the actual duration of the DS could be much longer than reported.

### 3.2 Spatial Variations of Dust Storms

**Figure 2:**
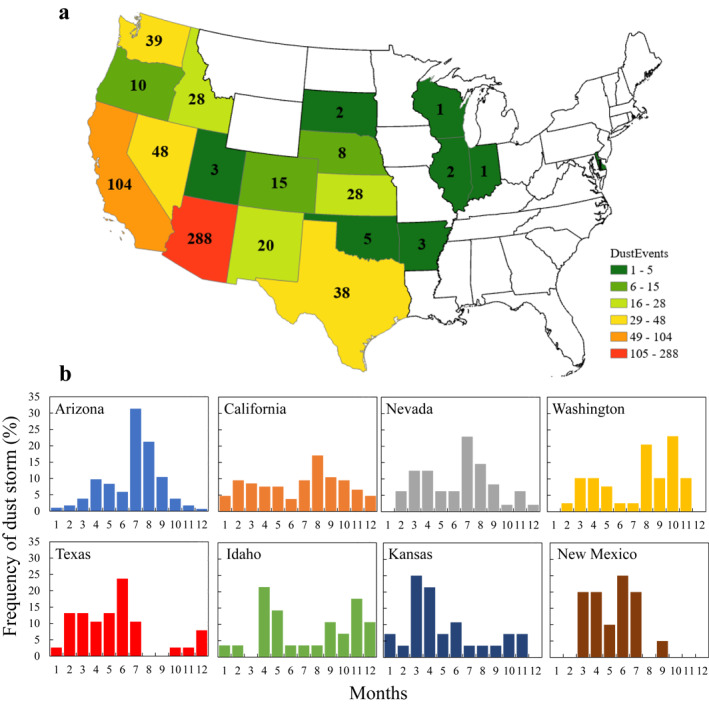
(a) Distribution of dust storm (DS) events (after removing duplicated reports) per state, color indicates the number of DS reports. (b) Monthly distribution of the DS events per state as reported in the SED, for states that had more than 20 DS reports in total.

A total of 21 states had reports of DS in the SED between 2000 and 2020 (Figure 2a). Some states (11 in total) had less than 10 DS reports, while others (10 in total) had multiple reports ranging from 10 (Oregon) to 288 (Arizona) in total. While a majority of the reports are in the western part of the country, several states in the central (e.g., Wisconsin, Indiana, and Illinois) or eastern (e.g., Delaware) part of the USA had also reports of DS. Next, we looked at the monthly distribution of DS reports in states that had more than 20 DS reports in total (Figure 2b). Monthly distribution showed that some states had a higher frequency of DS in the summer while others were most prevalent in the spring.

### 3.3 Economic Costs and Fatalities/Injuries Reported From Dust Storms

The SED is known to be one of the most commonly used data sources that examine hazard losses (Black & Mote, 2015). According to the SED guidance (NWS, 2022b), Direct Fatalities/Injuries resulting from DS would be people who were asphyxiated due to high dust/sand content in the air (rare), people who were hit by flying debris, or fatalities and injuries resulting from a vehicle being tipped/pushed over or blown off a road by the strong winds, resulting in an accident and associated fatalities/injuries. Indirect fatalities/injuries from DS would be caused by vehicular accidents caused by reduced visibility during a DS or by debris left on a road after a DS passed. It should be noted that the SED guidance NWS (2022b) indicated that it is hard to determine whether a fatality or injury was caused by a direct or indirect reason, and therefore should be examined on a case‐by‐case basis.

Since the SED provided information on DS events that were associated with deaths, injuries, and material losses (properties and crops), an examination of the reported number of deaths and injuries (direct and indirect), as well as property and crop losses, was performed (Table 1). The highest number of deaths (12) were reported in 2009, with 5 direct and 7 indirect deaths. The highest number of injuries (71 all direct) were reported in 2003. The property losses ranged from 0 in 2008, which had the lowest yearly number of DS, to $2,290,000 in 2013 which had 42 DS reports (but not the year of greatest number of reports). No strong correlation (low *R* ^2^ values) was found between the total number of DS per year and the number of deaths, injuries, or material losses, but a positive slope was observed for all three (deaths, injuries, and material losses, data not shown). It should be noted that less than 7% of the DS events had reports of injuries, while less than 3% had reports of deaths. We also noticed that several DS events had in their episode narrative, which is part of the database, reports of injuries (e.g., “One motorist was injured in a weather related accident along U.S. Highway 84 in Garza County”), or death (e.g., “Reduced visibility contributed to multiple vehicle accidents on I‐70 which resulted in one fatality and nine injuries”) but no reports (counts) of injuries or death in the database. Some of the episode narratives also reported damages (e.g., “Damages were estimated to exceed $350,000 across the region”) but no monetary valuation was provided for the material losses for the event.

**Table 1:** Annual Number of Dust Events, Counts of Deaths, and Injuries (Direct and Indirect) As Well As Property Losses, and Crop Losses Based on Dust Storm Database From 2000 to 2020

Year	Number of events	Direct injuries	Indirect injuries	Direct deaths	Indirect deaths	Damage to properties^a^	Damage to crops
2000	18	29		1		$190,000	
2001	18	5				$180,000	
2002	35	45		2		$427,000	
2003	30	71		2		$284,000	
2004	22	11				$80,000	
2005	15	32				$70,000	
2006	29	22		2		$690,000	$2,250,000
2007	28	4	3		2	$950,000	
2008	12						
2009	32		56	5	7	$760,000	$5,000,000
2010	25		7		1	$140,000	
2011	33	4	50		2	$848,000	
2012	47		35		1	$1,450,000	
2013	42		68		6	$2,290,000	
2014	45	16	14		3	$793,000	
2015	29	15		2	1	$25,000	
2016	45	1	17	3	1	$1,292,000	
2017	29		9		3	$345,000	
2018	53	5	3		3	$900,000	
2019	22					$100,000	
2020	38	6	18	1	2	$512,000	

^a^ Amount in $ represents value for time of report.

### 3.4 Limitations of the Storm Events Database

**Figure 3:**
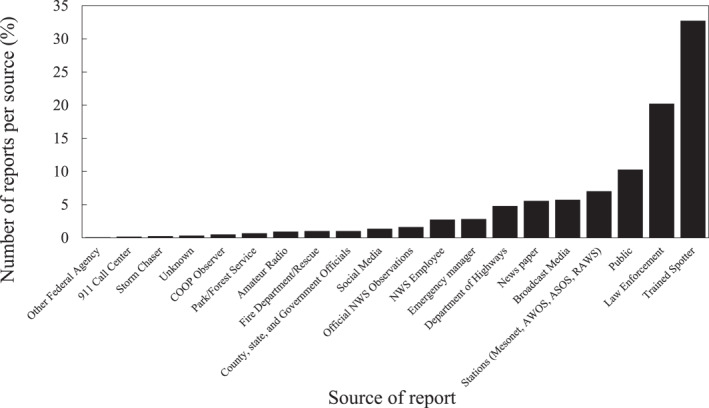
The frequency of each of the sources reporting dust storm events in the SED.

An examination of the reporting source of the DS events in the SED (Figure 3) shows that the sources of the reports vary from professional and trained personnel to automated reports by an ASOS station to reports from the public. The greatest percentage of the DS events (34%) was reported by trained spotters and the next most frequent reports were from law enforcement (19%). Many DS events had multiple reports (up to 29) from different sources, creating an oversampling issue that becomes problematic if this dataset is used to derive long‐term dust trends.

Since this is the only known nationwide database for DS events in the USA it was impossible to compare or verify all the DS events. Therefore, we decided to explore each of the 647 DS reported. First, we identified the location of the events in the SED based on the county and the episode narratives which may provide detailed information (e.g., “Interstate 10 near Benson,” “reported in Casa Grande, Florence,” etc.). Next, we examined all the ASOS in the state with the DS report with a focus on the ASOS that was closest to the location reported. The meteorological conditions from the ASOS were examined to identify each dust event with wind speed and weather code (if available). Next, we examined the visibility values during the time reported, if the horizontal visibility was below 1 km the event would have been defined as a DS, if the visibility was >1 km it would not be defined as a DS but as BLDU.

Out of the 647 DS reports in the SED, 156 could not be examined as there was no ASOS near the location reported or the ASOS was not operational at the time of the DS event, leaving 491 reported DS that could be examined. Out of the 491 DS reported in the SED, 187 events (38.1% of the 491) were confirmed as dust storms and had visibility below 1 km, and 59.9% of these 187 DS had visibility <0.4 km. In addition, we noticed that 10 of these DS in the SED were reported in one place (that did not have a reduction of visibility) but the DS was observed and identified in another location in the state during the same time. The remaining 304 DS reports contained 57 events that did not have any documented reduction of visibility (across the state) although strong winds were observed, and 245 (49.9% of the 491 DS reported in the SED) should have been defined as BLDU events, as the visibility was reduced but it was >1 km. We also noticed that one DS event reported in the SED was a dust devil. Almost all the states examined had BLDU events that were inaccurately listed in the SED as dust storms or other reported DS that could not be examined or meet the 1 km threshold to be defined properly as DS. By examining each of the DS, we noticed that one of the DS from New Mexico that had the longest duration reported (1 April to 9 April 2011) seems to have a mistake in its start date included in the SED.

To highlight some of these findings several locations were examined to present issues with the accuracy of the SED reports. These locations include several locations in Utah, Denver, and La Junta in Colorado, Lubbock and El Paso in Texas, as well as the Greater Phoenix area in Arizona, see maps in Figure S2 in Supporting Information S1 for the ASOS used in each location. There were three DS events reported for Utah from 2000 to 2020 in the SED: the first one was on 12 June 2003, at Thompson Springs. This event was reported by law enforcement. Since the nearest ASOS unit was >30 km from Thompson Springs we could not examine or confirm this event. This was the case for 156 DS events (24% of the DS reported in the SED). The next DS event examined for Utah was on 7 August 2006, at Provo. The visibility value from the nearest ASOS unit (PVU) at Provo was as low as 4 km (not meeting the DS criterion) and no weather code was reported. This was the case for 245 DS events that were reported in SED. The next DS event was reported by an official NWS observer on 7 June 2006, at Hanksville. The ASOS unit 4HV located at Hanksville reported a DS on the same day when the visibility was reduced to 0.8 km, confirming the SED report. Similar observations were made for an additional 186 DS events. But when we examined the 4HV ASOS unit for the entire period we notice it had another DS (reported as BLDU) on 14 April 2009, that had a visibility of 0.4 km, which was not reported in the SED. This raised the possibility that there were additional DS that occurred across the nation that were not reported in the SED. Therefore, we examined the SLC ASOS unit (Salt Lake City International Airport) that did not have any reports in the SED from 2000 to 2020. We found three DS dates (3 March 2010, 22 April 2014, and 14 April 2015) that had visibility below 1 km (0.8 km), yet none of these days were reported in the SED. The 14 April 2015 event was notable as it had visibility below 0.4 km, caused a fatal accident on the highway and significant damage, and was widely reported both in the news media (Alberty & Mims, 2015) and the scientific literature (Nicoll et al., 2020). Another Utah DS event that was not reported in the SED, on 15 April 2002, was also reported in the literature (West & Steenburgh, 2010). Observation of all ASOS units from Utah during this DS event showed that six different ASOS units recorded visibility lower than 1 km, with three even recording visibility lower than 0.4 km, making it unclear why this DS instance was not reported in the SED.

A similar analysis was performed for Colorado which had 15 DS events in the SED, but only seven of them could be examined with an ASOS near the reporting area. Two events were confirmed as DS and five were occurrences of BLDU with visibility that did not meet the DS criterion. Based on these findings we decided to examine two ASOS units in Colorado from 2000 to 2020 (see Figure S2 in Supporting Information S1 for location), the first was Denver International Airport which did not have any reports in the SED. We found two DS events that were not reported in the SED (20 February and 22 October in 2001) that had visibility below 1 km (0.8 km). Next, we examined the LHX ASOS site (La Junta Airport in Otero County); this location had one report in the SED on 11 January 2003. After examining the ASOS for the entire duration we found 20 additional DSs at La Junta Airport. One of these events (on 29 April 2014), which had visibility of 0.4 km, was reported in the SED for two other counties (Cheyenne and Kit Carson counties) but was not mentioned for LHX or Otero County. Texas had a total of 38 DS reported in the SED; 12 of these dust storms could not be confirmed as they were reported in locations that did not contain an ASOS or the ASOS was not operational at the time of the DS event. Out of the remaining 26 DS reported in the SED, 14 (36.8%) were confirmed as dust storms and 12 (31.6%) had visibility >1 km and thus did not meet the DS criterion. Next, we performed an analysis of ASOS in two locations, Lubbock and El Paso (see Figure S2 in Supporting Information S1). Using data from our previous work (Kelley & Ardon‐Dryer, 2021) for Lubbock, Texas we were able to examine this location in depth. A total of 14 different DS were reported in the SED for Lubbock from 2000 to 2020. One of those events did not have a record in the LUB ASOS unit. Observations of the lowest visibility values from each of the reported events showed that 46% of them had visibility >1 km (range 1.2–4.8 km). If visibility observations would have been made based on the NWS (2022a; 2022b) criterion for a DS (<0.4 km) then 69% of the reported DS in the SED for Lubbock should have not been characterized as such. One DS event that was reported in the SED for Midland but not for Lubbock, occurred on 15 December 2003. This event, which had visibility lower than 0.4 km in LUB ASOS, was also reported in the literature (Lee et al., 2009) but was not reported in the SED for Lubbock. The 22 January 2012 DS in Lubbock was also not reported in the SED and yet it had visibility lower than 1 km, qualifying as a DS and was presented in the literature (Kandakji et al., 2020). Next, we explored all the LUB ASOS data (2000–2020) to examine if there were additional DS that were missed from the SED. We identified a total of 26 DS reported by the in Lubbock ASOS during this period with that had visibility <1 km, 10 of them with visibility <0.4 km. A total of 20 (77%) of these DS events were not reported in the SED. We were surprised there were no DS reports in the SED for El Paso, as we know it experiences frequent dust events (Kandakji et al., 2020; Novlan et al., 2007; Rivera Rivera et al., 2009, 2010). The ASOS unit at El Paso International Airport was examined from 2000 to 2020, and any dust storms that occurred during that period were recorded. We found a total of 36 dust storms for El Paso, yet none of them were reported in the SED. This finding emphasizes the underrepresentation of DS events in the SED.

Following our findings in Tong et al. (2022), where we examined a portion of the DS reported in the SED from the Phoenix area that was used by Comrie (2021), we decided to find DS reported by ASOS for the greater Phoenix area from 2005 to 2020 (utilizing an existing database; Ardon‐Dryer et al., 2021), using an analysis of nine different sites (see Figure S2 in Supporting Information S1 for ASOS information and locations). A total of 87 dust storms were found from 2005 to 2020. When we compared this DS list to the one reported in the SED, we found that 40 of those DS events in the SED were included and had visibility <1 km, but an additional 47 DS events (54%) which occurred in the greater Phoenix area were missing from the SED dataset. There were additional 27 DS reported in the SED for the greater Phoenix area, which did not meet the DS criterion of visibility <1 km.

Previous studies have used data from the SED to analyze the reporting of other various weather phenomena (Ashley & Black, 2008; Bentley et al., 2002; Dixon et al., 2005; Markowski et al., 1998). Many found the SED to be an inconsistent and inaccurate record of severe weather (Ashley & Black, 2008; Ashley & Gilson, 2009; Black & Ashley, 2010; Black & Mote, 2015; Blair et al., 2011; Downton et al., 2005; Miller et al., 2016; Trapp et al., 2006). Others also found the reports on damage, injuries, and fatalities to be incomplete and inconsistent (López et al., 1993; Santos, 2016). Shoemaker and Davis (2008) presented discrepancy issues with SED deaths and injuries data for Arizona, showing an event where the SED reported no deaths, yet other records reported 23 deaths during the same event. Lader et al. (2016) indicated that the Arizona Department of Transportation reported at least 29 incidents that had 1 or more fatalities due to dust events in Arizona during 2000–2011, yet the SED only reported 12 fatalities in total for the same period in Arizona. Comparison based on the total number of injuries and fatalities from the SED for Arizona for the period of 2000–2011 to those presented in Lader et al. (2016) showed that the SED report underestimated the number of direct and indirect injuries and fatalities by 7.6 and 5.7 times, respectively.

Some studies that have examined the impact of DS on human health highlighted some of the issues with the SED. For example, Crooks et al. (2016) mentioned that the NWS does not guarantee the accuracy of the information in the database, and sometimes the only location information provided is the NWS weather forecast zone in which each storm is reported. Rublee et al. (2020) noted that there were areas known to experience DS activity, but they were not reported in the SED. Jones (2020) also mentioned that there could be issues with the databases, mentioning that there is a possibility that some dust storms, especially those that were short and low‐intensity in rural areas were not included in the SED. Another issue suggested by Shoemaker and Davis (2008) indicated that there is a significant bias in the number of reports along roadways in predominantly rural areas or at high‐density population centers, which may reflect only a subset of these events. Also, the reports are based on observations which might be biased to daytime, causing under‐reporting of DS that occur at night. We noticed that 25% of the dust storms not reported in the SED in the greater Phoenix area occurred after 20:00 local time. In some locations we noticed that days that had two dust storms on the same day, had the daytime event reported in the database but not the nighttime one (e.g., DS in San Diego California on 4 September 2003).

Our analysis shows that there are many issues with reporting dust storms in the SED, with the inclusion of many dust events that do not meet the criteria for a DS (having visibility >1 km) along with an underrepresentation of events that do fit the criteria needed for a DS but are not included in the SED. Peterson and Zobeck (1996), who used the Storm Data publication to examine the record of DS events in the western US from 1972 to 1992, stated that some of the patterns found were plausible, some were puzzling, and some were perhaps dubious, finding an under‐reporting of dust storms in the Lubbock area in comparison to their published analysis (Wigner & Peterson, 1987). Peterson and Zobeck (1996) stated that many of these events may not have been noted outside of the region. Furthermore, they stated that the occurrence of dust in the Lubbock area may not have seemed sufficiently extraordinary to warrant reports to be submitted to the SED. Rublee et al. (2020) stated that due to errors or biases in reporting DS events in the SED, the number of DS reported to the NWS may not represent the true number of dust storms that occurred in the USA.

We suggest that these issues could be caused by multiple factors. There is potential confusion in the reporting protocols for DS events as the NWS (2022b) states that dust storms that occur in direct relation to convection should be entered as a thunderstorm wind event, including the appropriate wind magnitude, not as a DS entry; but when a DS moved away from the parent thunderstorm or convection and presents as its own hazard or threat, it should be classified as a DS event. These definitions seem confusing (especially to non‐meteorologist contributors to the SED, such as law enforcement officers), as “a dust storm is a dust storm” and should be reported when it reduces the visibility below the threshold regardless of whether it was created with or without a thunderstorm. Similar rule‐based under‐reporting of dust storms associated with thunderstorms in Australia was lamented by O’Loingsigh et al. (2010). This cannot fully explain the under‐reporting of dust storms in Lubbock noted in our analysis and by Peterson and Zobeck (1996), as most of the dust storms (>60%) were not caused by convective thunderstorms and should have otherwise been reported.

An additional factor could be attributed to the wide range of sources used to report DS events (Figure 3). Storm Events Database reports can originate from non‐trained/non‐meteorologist human observers such as law enforcement and the general public (Miller et al., 2016). According to the SED guidance, 40 different sources can provide reports to SED (NWS, 2022b). Previous studies questioned the fact that some of the database population was gathered from media reports via newspaper and clipping services (Ashley & Gilson, 2009; Peterson & Zobeck, 1996). Edwards et al. (2018) stated that the SED does not contain information on the quality of the report nor on the experience level of the entity making these reports. We found events that were reported by trained spotters that should have received instructions to report dust storms only if the visibility was <0.4 km following the U.S. Department of Commerce (2011) guidance, yet these reported dust events had visibility (>1 km) that did not meet DS criteria. Miller et al. (2016) stated that the quality of the reports in the SED is not guaranteed even though the NWS attempts to use the most accurate information available. The Storm Data preparation document indicates that the NWS puts an effort to use the best available information, but because of time and resource constraints, information from sources outside the NWS may be unverified by the NWS, indicating that the NWS does not guarantee the accuracy or validity of the information (NWS, 2022b). However, the data gathered in the SED have significant impacts on policy, mitigation, and resource allocation and are widely used by scientific researchers in fields outside meteorology (e.g., Cutler, 2015; Jones, 2022) as well as in forensic investigations (Grimshaw & Ploger, 2018), public health assessments (Lothrop et al., 2022; Rublee et al., 2020), economic analyses (Griffin et al., 2021), finance (Bourdeau‐Brien & Kryzanowski, 2019) emergency planning (Hays County Texas, 2017) and legal and political matters (Sisco, 2021). Therefore, the accuracy and precision of the database should be of great importance.

The SED performs a valuable public service to many user and stakeholder communities in assembling data from across the USA on daily weather events of an impactful nature, and we appreciate the importance and utility of the database, as well as the difficulty in assembling it. We believe that because it is so widely used by professionals in diverse fields, accuracy should be the ultimate goal, therefore there should be greater emphasis on improving the methodological foundation of this database. There is a need for a more formal, efficient, precise and accurate way to collect the storm data, as well as to validate and verify the reports in the SED. Investigators in many different fields need to know that the data in the SED, while representative, may not be comprehensive: the data are useful, but not necessarily accurate, and thus caution is warranted in using *Storm Data* database for quantitative studies of dust storms (or other impactful weather phenomena) and their effects.

There appears to be a lack of consistent definitions and reporting of “dust storm” and of dust weather in general in the USA, as well differing understanding of the meaning of the term “dust storm” by persons outside atmospheric science. What is more, consistent definitions of “dust storm” have not been used by those investigating the climatology of dust weather and the health, safety, and other societal effects of dust across the USA. This potentially limits the inter‐comparability of these and other studies to each other and to analyses of dust occurrence in other nations, and also limits understanding their associations with human health and safety and societal impacts. In addition, since the USA experiences far more events of BLDU (visibility >1 km) than it does dust storms, we note the need for a new database that will include dust events of all types including BLDU and dust storms.

## 4 Conclusions

This study examines the long‐term variations of reported dust storms from the SED and its reliability and usefulness as well as limitations as a source for documenting the occurrence of dust storms across the USA. While this database provides a large amount of useful information for understanding the frequency, distribution, importance, and impacts of significant weather across the USA, including dust storms, our analysis of events occurring from 2000 to 2020 shows that it is lacking many dust storms that occurred during that period, and also contains many events of BLDU (with visibility >1 km) that should not have been reported as dust storms. There are also multiple entries in the database from various sources for a single DS that could be problematic if the number of entries in the dataset is used to derive long‐term dust trends. Some of the causes for the underrepresentation of DS events or confusion in reporting in the SED could be attributed to the diverse sources contributing to the reports, potential lack of consistency in reporting criteria, and lack of verification of the reports. Although it is one of the most widely used dust databases and impactful weather available for the entire USA, the issues found in this study raised questions on its, accuracy, and reliability as a dataset to study dust climatology and associated health and socioeconomic effects. Therefore, we recommend that those using the SED or *Storm Data* for health, safety, and other studies will use it with caution and understand its potential limitations which might affect the findings of their research.

## Conflict of Interest

The authors declare no conflicts of interest relevant to this study.

## Supporting information

Supporting Information S1

## Data Availability

All the dust storm data used in this study were downloaded from the National Center for Environmental Information Storm Events Database (https://www.ncdc.noaa.gov/stormevents/). Automated Surface Observing Stations data were retrieved from Iowa State University's Iowa Environmental Mesonet (https://www.mesonet.agron.iastate.edu/request/download.phtml).
