# Supplementary material for: When a Dust Storm Is Not a Dust Storm: Reliability of Dust Records From the Storm Events Database and Implications for Geohealth Applications
Source: Geohealth. 2023 Jan 5;7(1):e2022GH000699. doi: 10.1029/2022GH000699 (PMC9813799; doi:10.1029/2022GH000699)
Supplement: Supplementary file 1 — Supporting Information S1 [file GH2-7-e2022GH000699-s001.pdf]

When A Dust Storm Is Not A Dust Storm: Reliability of Dust Records from the Storm Events Database and Implications for Geohealth Applications

K. Ardon-Dryer[1], T. E. Gill[2], and D. Q. Tong[3]

[1] Department of Geosciences, Texas Tech University, Lubbock TX, USA 79409
[2]Department of Earth, Environmental and Resource Sciences, The University of Texas at El Paso, El Paso, TX, USA 79968
[3]Department of Atmospheric, Oceanic and Earth Sciences / Center for Spatial Information Science and Systems, George Mason University, Fairfax, VA, USA 22030

Contents of this file
Figures S1 and S2

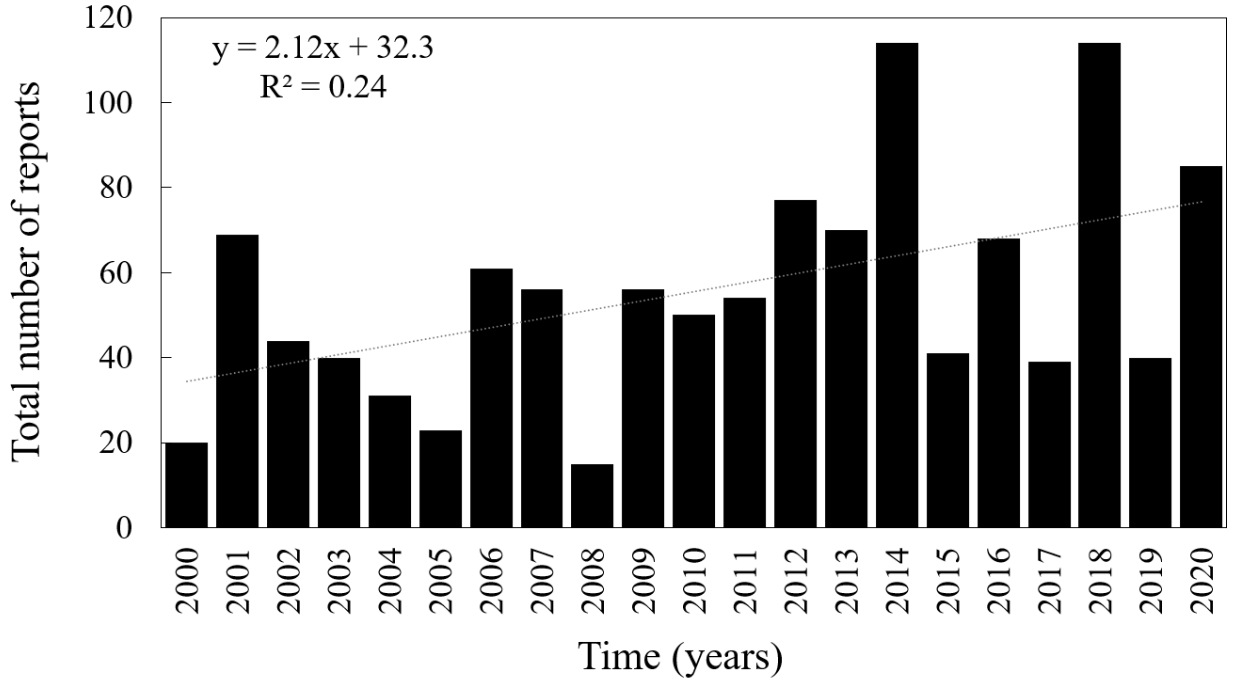

Figure S1. Number of dust storms reported per year.

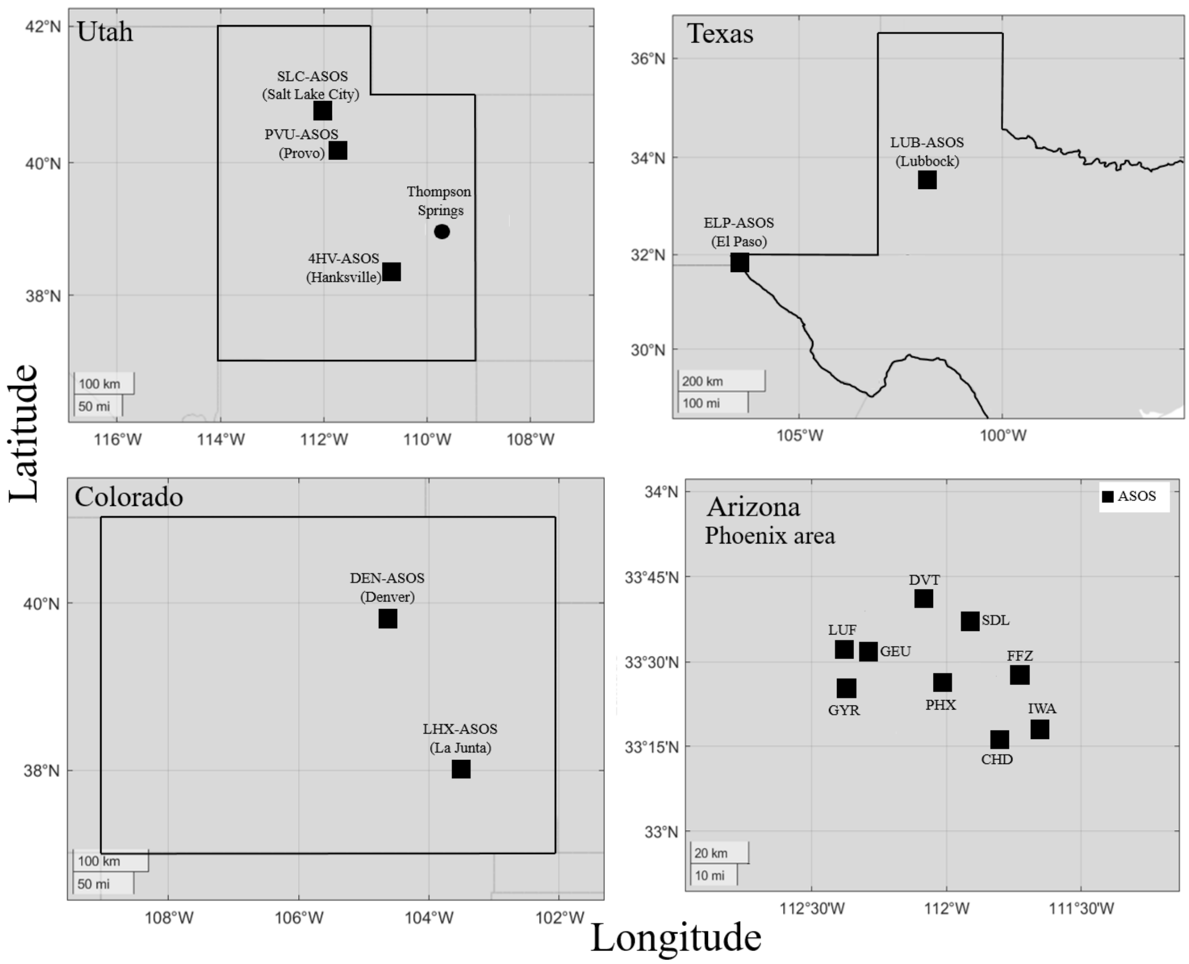

Figure S2. Maps for ASOS from different locations examined as described in the text.
